# Effect of Sequential Kicks on Programming Time and Movement Time in Taekwondo

**DOI:** 10.5114/jhk/194067

**Published:** 2025-06-25

**Authors:** Chung-Yu Chen, Ching-Hui Yu, Ti-Yu Chen, Tai-Yuan Su

**Affiliations:** 1 Department of Physical Education, National Taiwan University of Sport, Taichung, Taiwan.; 2 Department of Sports, National Changhua University of Education, Changhua, Taiwan.

**Keywords:** response complexity, round kick, reaction time, combat sport

## Abstract

It is a critical tactic in combat sports to launch consecutive attacks that consist of two or more individual kicks strung together in time. The study aimed to assess how the number of round kicks would impact reaction time (RT) and movement time (MT). Twenty-eight experienced taekwondo athletes performed one of four different kick responses randomly in a simple RT paradigm: 1) clenched lead fist without a kick (task A); 2) round kick following task A (task B); 3) double kick following task B (task C); and 4) turning kick following task C (task D). Electromyography recordings from the thenar muscle and acceleration impulses from a triaxial accelerometer positioned at the heavy bag were used to determine premotor time and kick time, respectively. Repeated-measures ANOVA revealed that task B, task C, and task D significantly increased RTs (p < 0.001). The RT of task D was longer than that of task B and task C (p < 0.001). The movement time of the round kick in task C and task D was longer than in task B (p < 0.001). The movement time of the double kick in task D was longer than in task C (p = 0.003). Additional time is required to prepare for multi-kicks responses. An increased number of individual kicks to execute as a whole may slow down the kick movement.

## Introduction

Combat competitors need to execute reactive or self-initiated movements during a match (Martinez de Quel and Bennett, 2014). Efficient attacks, depending on competition rules, can earn one or more points, ultimately determining the winner or the loser in each round or contest through the total points or a knockout. This impels a competitor to adopt tactics such as quick starts, feints, attacks or counter-attacks to accumulate points (Tornello et al., 2014). Additionally, besides individual attack or defense movements, combination movements are also critical for point accumulation (Davis et al., 2018; Tornello et al., 2014). Previous research has reported the kinematics or kinetics of single technique in combat sports such as taekwondo (Diniz et al., 2021; Moreira et al., 2016), boxing (Lenetsky et al., 2020; Loturco et al., 2016; Piorkowski et al., 2011), karate (Diniz et al., 2021; Martinez de Quel and Bennett, 2014), and judo (Ishii et al., 2018; Prończuk et al., 2023, 2024, 2024a). These studies required movements to be executed as swiftly and/or powerfully as possible, closely simulating real competitive condition. Independent variables such as the subject’s skill level, target settings, combat sports modality, and others were manipulated to assess the effects of selected techniques on kinematic and kinetic outcomes. The reaction time (RT) and movement time (MT) of single taekwondo kicks (Ervilha et al., 2020; Sant'Ana et al., 2017) and single karate punches (Martinez de Quel and Bennett, 2014) were notably shorter for experts compared to novices.

Reaction time is the measurement of time latency between the stimulus onset and movement initiation, representing the time required for stimulus identification, response selection, and response programming in movement preparation (Schmidt et al., 2019). In a simple RT paradigm, participants are instructed to respond to a single go-signal as quickly as possible with a specified movement. Reaction time tends to increase with the complexity of responses that involve multi-element movements (Henry and Rogers, 1960). The classic experiment by Henry and Rogers provided evidence supporting the notion that simple RT latency is necessary for planning and initiating the responses, contributing to the theoretical model of information processing (Henry and Rogers, 1960; Klapp and Maslovat, 2020). Previous studies have reported that increasing response complexity, defined as the number of elements involved, may lead to a longer preparatory process requirement for initiating tasks such as manual button presses (Maslovat et al., 2018, 2019) and speech articulation (Klapp, 2003). These earlier investigations focused on planning times in laboratory tasks, with participants either allowed or not allowed to practice them. In the present study, we recruited elite taekwondo athletes who could accurately perform the required kick movements. Participants deliberately practiced a series of round kicks in every training session, including kicks with the lead and the rear foot, double kicks, turning kicks, and kick combinations. Manipulating combination movements of taekwondo kicks in the context of simple RT and MTs may provide valuable insights into the increased response programming time during continuous offense and its impact on the risk of being counter-attacked.

Despite extensive research on the kinematics and kinetics of single techniques in combat sports, such as taekwondo, boxing, karate, and judo (Diniz et al., 2021; Moreira et al., 2016; Lenetsky et al., 2020; Loturco et al., 2016; Piorkowski et al., 2011; Ishii et al., 2018), a significant knowledge gap exists regarding the complexity of sequential attacks. Most studies have focused on the performance of isolated techniques, examining variables such as a skill level, target settings, and sports modality, but they have not addressed the intricacies of executing combination movements. Furthermore, while RT and MT have been well-documented for single taekwondo kicks and karate punches (Ervilha et al., 2020; Greco et al., 2024; Martinez de Quel and Bennett, 2014; Sant'Ana et al., 2017), there is a lack of research on how these metrics change when multiple offensive movements are performed in succession. Therefore, the purpose of this study was to examine whether the simple RT and kick duration were influenced by the number of offensive movements in a sequence. Building upon previous research, we hypothesized that 1) assessments of sequential taekwondo kicks would exhibit significantly slower simple RT compared to a single offensive movement, and 2) the duration of movement time would be adversely affected by the subsequent kick movements.

## Methods

### Participants

The experimental protocol and procedures were approved by the Institutional Review Board at the Tsaotun Psychiatric Center (protocol code: 104001; approval date: 04 April 2015). Participants received information about the procedures and risks of the experiment prior to their inclusion in the study. Twenty-eight highly experienced taekwondo athletes (14 males and 14 females) participated, with an average age of 20.0 ± 1.4 years, body mass: 62.4 ± 13.2 kg, body height: 1.70 ± 0.01 m, and training experience: 12.0 ± 2.8 years. All participants were in good physical condition and were excluded if they reported musculoskeletal injuries, infections, or risky cardiopulmonary factors that would impair their kicking abilities. They had at least six years of taekwondo training experience, were national-level athletes, and had won at least one medal at the National Games or National Intercollegiate Athletic Games. Nineteen of them had also competed in the Asian Games, the Universiade or the world championship either before the research began or within one year after its completion. Some of these nineteen athletes had even won medals in these international competitions.

### Measures

The kick target consisted of two cross signs positioned on the sides of a heavy hanging bag (1.60 m × 0.7 m and weighing 90 kg), with both crosses fixated at a height of 1.20 m above the ground. A triaxial wireless accelerometer (Noraxon Inc., Scottsdale, AZ, USA; ± 24 G) was positioned at the anterior of the heavy bag, extending vertically 1.20 m from the ground. A light-emitting diode (LED) was mounted on 1.60 m above the ground and at the anterior of the heavy bag to generate the go-signal for kicking. Surface EMG, acceleration and go-signal data were recorded at 1500 Hz using a direct transmission system with a USB receiver (TeleMyo DTS, Noraxon Inc.). The electrodes used were 2.5 cm round Ag/AgCl electrodes (BlueSensor, Ambu Inc., Ballerup, Denmark), and were adhered to the thenar muscles of the lead arm.

### Design and Procedures

**Figure 1:**
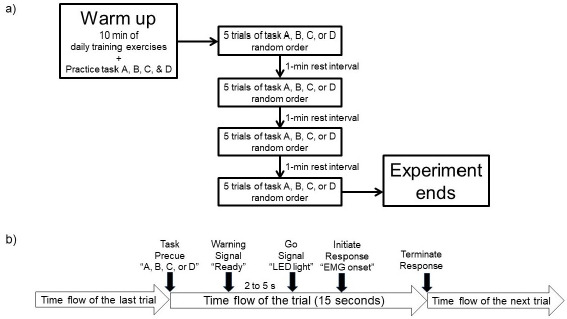
Schematic presentation of the study design. a) Overview of the experimental procedure. b) Timeline of events in one trial.

Participants performed four responses in a simple RT paradigm: task A involved simply clenching the lead fist immediately after the go-signal (LED stimulus) was presented; task B required clenching the lead fist immediately after the LED stimulus was presented, followed by a round kick with the lead foot; task C involved executing a double kick following task B; and task D required performing a turning kick following task C. The task processes are demonstrated in the Youtube video available at (https://www.youtube.com/watch?v=Tdo5kYEZpQ8). The experimental session involved 20 trials, with 5 for each of the four tasks, scheduled in random order. Before the tests, participants were allowed to warm up, which included 10-min daily training exercises and practice trials performed at minimal effort. Next, the skin was cleaned using an alcohol wipe to prepare for the placement of the EMG electrodes. The EMG electrodes were checked for adequate adhesion by observing the consistent baseline signal during repeated rapid clenched fist movements. Figure 1a provides an overview of the procedures used.

Each response began with participants standing in a ready stance at a self-selected execution distance from the heavy bag to promote maximal performance. Participants were informed prior to each trial about the type of response they would execute. They received the verbal instruction “ready” before the LED lit up and were instructed to react as quickly and forcefully as possible by the clenched fist or kicking the target after clenching their fist. The foreperiod, or the time between the “ready” sound and the go-signal, ranged from 2 to 5 s, and the inter-trial interval was fixed at 15 s using a stopwatch (Figure 1b). Participants were also instructed to return to the ready stance after completing the response tasks. Although the heavy bag was quite heavy and stable, an experimenter stood at the posterior side of the heavy bag to ensure stability and to inspect the achieved height of the required kick in this study.

### Data Reduction

**Figure 2:**
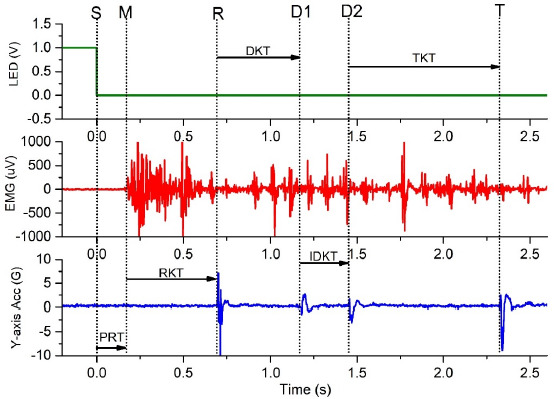
Example representation of the experimental signals of task D. Note: Dotted line S indicates the LED stimulus generated; dotted line M, the starting point of the thenar muscles activation; dotted line R, the contact point of the round kick; dotted line D1 and D2, the first and the second contact point of the double kick, respectively; dotted line T, the contact point of the turning kick. PRT = premotor time; RKT = movement time of the round kick; DKT = movement time of the double kick; IDKT = intra-kick time for the double kick; TKT = movement time of the turning kick

Raw EMG data were band-pass filtered from 10 to 450 Hz using a Butterworth digital filter. The starting points of the EMG onset and the impact points of kicks were determined from the filtered EMG and the Y-axis accelerometer, respectively, when they exceeded baseline value by more than three standard deviations. All signals were processed and extracted using a custom MATLAB program (Math Works, Natick, MA, USA). The premotor time (i.e., simple RT) and kicks time (i.e., MTs) were determined as shown in Figure 2: (1) premotor time (PRT) was calculated from LED stimulus generation to the EMG onset of the thenar muscles; (2) movement time for the round kick (RKT) was calculated from the EMG onset to the first target contact; (3) movement time for the double kick (DKT) was calculated from the first target contact to the second target contact; and (4) intra-kick time for the double kick (IDKT) was calculated from the second target contact to the third target contact. The fourth target contact on the Y-axis accelerometer signal was used to inspect the movement completion of the turning kick, and the time of the turning kick was calculated from the third target contact to the fourth target contact. The validity of pre-motor time was set to be above 100 ms and below 500 ms, which was deemed to result from a simple reaction. No outliers were present in the data.

### Statistical Analysis

The trials from each simple RT assessment and response condition were separately averaged to produce mean values. Descriptive data were presented as means and standard deviations. The intra-class correlation coefficient (ICC) was employed to assess reliability using five trials of each task for all participants. Data were analyzed using a one-way ANOVA repeated measures design to compare variables among or between the response tasks and to compute partial eta squared (*η*^2^_p_) and observed statistical power (SP) of the test. Subsequent post-hoc comparisons of means utilized the Tukey’s HSD procedure. The alpha level was set at 0.05 in all statistical analyses. The benchmarks to interpret the *η*^2^_p_ effect size were operationally defined as 0.01, 0.06, and 0.14, corresponding to small, medium, and large effects, respectively (Cohen, 1988). Statistical analyses were performed using SPSS Statistics for Windows (version 20; IBM Corp., Armonk, NY, USA).

## Results

The mean values for the premotor time and MTs of the four response tasks are presented in Table 1. Regarding the relative reliability of the different premotor times for the four response tasks, the ICC of each response exceeded 0.90 (ICC_task A_ = 0.90, ICC_task B_ = 0.97, ICC_task C_ = 0.90, and ICC_task D_ = 0.95), indicating excellent agreement (Donner and Eliasziw, 1987).

The means of premotor time exhibited significant differences, *F*(3, 81) = 23.59, *p* < 0.001, *η*^2^_p_ = 0.466, SP = 1.00. Tukey’s post-hoc tests revealed longer premotor time values for tasks involving two (task B), three (task C), or four (task D) sequential movements compared to the response of a single movement (task A). The response involving four sequential movements had a significantly longer premotor time compared to tasks involving two and three sequential movements. No significant difference was found between two and three sequential movements in premotor time.

A significant difference in movement time of round kicks was observed, *F*(2, 54) = 21.93, *p* < 0.001, *η*^2^_p_ = 0.448, SP = 1.00. Tukey’s post-hoc tests revealed that the single round kick (task B) was the fastest in comparison to the round kick that still required performing two or more sequential movements (task C and task D). There was no significant difference between task C and task D in the movement time of round kicks.

Regarding the first kick in the double kick movement, the time for task C was shorter than that for task D, which still required performing the turning kick, *F*(1, 27) = 10.57, *p* = 0.003, *η*^2^_p_ = 0.281, SP = 0.88. Similarly, the time for the intra-kick in task C was shorter than that for task D, which still involved performing the turning kick, *F*(1, 27) = 52.21, *p* < 0.001, *η*^2^_p_ = 0.659, SP = 1.00.

**Table 1:** Means (*M*), standard deviations (*SD*), and analysis of variance (ANOVA) results for premotor time and movement time of kicks (unit: ms) for each response task.

Variable	Task A	Task B	Task C	Task D	*F*	*p*	Post-hoc
PRT							
*M* *SD*	148.7 23.1	174.0 37.5	176.7 38.2	190.6 35.7	23.59	< 0.001	A < B, C < D
RKT							
*M* *SD*		559.9 99.3	610.2 113.7	608.5 105.9	21.93	< 0.001	B < C, D
DKT							
*M* *SD*			465.8 42.1	483.8 54.2	10.57	0.003	C < D
IDKT							
*M* *SD*			250.6 29.1	268.6 30.7	52.21	< 0.001	C < D
TKT							
*M* *SD*				884.5 99.1			

Note: PRT = premotor time; RKT = movement time of round kick; DKT = movement time of double kick; IDKT = intra-kick time for double kick; TKT = movement time of turning kick

## Discussion

The purpose of this study was to examine multiple sequential combat movements and their timing initiation or execution through the use of a simple RT paradigm. Our purpose was based on the concept of the information processing, which requires time for movement preparation in order to organize and execute activities (Henry and Rogers, 1960; Klapp and Maslovat, 2020; Maslovat et al., 2018, 2019). Thus, the increasing number of sequential taekwondo kicks was expected to lead to an increase in simple RT. As expected, the results of this study supported our hypothesis that one and two more sequential kicks after the round kick exhibited slower simple RTs. Furthermore, one and two or more sequential kicks after the round kick elicited longer duration of movement time to perform the round kick. Additionally, the double kick that was followed by a turning kick clearly resulted in a longer movement time for the double kick, in comparison to the double kick that was viewed as the ending movement. This finding indicated that it took more time to preprogram and initiate the response sequence. Even highly experienced taekwondo athletes still needed more time to organize and execute the sequential kicks.

Reaction time is the time measurement from the onset of a go-stimulus until the required response emerges. It represents the transmission speed of the neural command or the time required for information processing to action. The increase in response complexity that delays the RT interval is consistent with previous literature that has reported the manual actions (Franks et al., 1998; Henry and Rogers, 1960; Maslovat et al., 2018, 2019) and the pronunciations (Klapp, 2003) in laboratory settings. These cumulative findings indicate that simple RT increases when there is a series of movements that an individual must perform. Complex motor movements were chosen based on previous work (Maslovat et al., 2018, 2019) to examine the distribution of processing in choice RT and simple RT paradigms. Klapp and Maslovat (2020) proposed that the timing code for a movement had to be compiled during the simple RT interval before the initiated response could be selected and performed. There existed a capacity limitation in working memory that restricted the storage of the timing code. The increase in simple RT was caused by the effect of the psychological refractory period, which ensured that sequential movements proceeded as desired or required before the go-stimulus onset (Klapp et al., 2019). The delay in the simple RT interval represented a bottleneck phenomenon for the brain to compile the timing code for an immediate action and to prevent the occurrence of the next action. The compilation of the timing code, paired with its corresponding response action, serves to prevent the occurrence of inappropriate actions. The process of compiling the timing code initiates the response during a simple RT task. This link between the compilation of the timing code and the initiation process as inseparable components of the simple RT interval is critical (Klapp and Maslovat, 2020; Maslovat et al., 2019). In this study, a simple RT task was used, and the findings showed that one or more sequential kicks significantly increased premotor time. However, no significant difference in premotor time was observed between task B (one kick after the clenched fist) and task C (two kicks after the clenched fist). These results supported previous literature that has demonstrated the time required to initiate the current movement for the compilation of the timing code with the limitation of working memory. The MTs of the round kick increased when they were followed by one or more kicks, indicating that additional time was needed to prepare and initiate the following kick. Moreover, the MTs of the double kick in the first and the second kick seemed to be influenced by the succeeding movement (turning kick). Although the round kick in the lead foot, the double kick and the turning kick all belong to the round family of kicks, the double kick and the turning kick still needed to be retrieved and compiled before initiation of the kick movement. Movement time latencies for round kicks under task C and task D, and for the double kick under task D, were evident in our results. These findings provide additional insights into the differences in processing time due to the factor of response complexity.

The round kick follows a proximo-distal sequence of inter-segment motion (Estevan et al., 2015) and represents a fundamental attack and counterattack technique utilized in sparring competitions across various combat sports, including taekwondo, wushu-sanda, karate, and muaythai sports. Rapid execution of this technique is essential for achieving successful scoring outcomes during matches. Sant'Ana et al. (2017) observed the effects of fatigue on premotor time (the time interval between visual stimulus and activation response of the rectus femoris) and execution time (the time interval between muscle activation and kick impact) of the round kick in taekwondo athletes. They reported an increase in RT after the fatigue protocol, but no difference in execution time. Additionally, Moreira et al. (2016) found that the premotor time of the gluteus maximus and the gastrocnemius lateralis was longer in the sub-elite athletes (over 200 ms) compared to elite athletes (less than 200 ms) during the roundhouse kick. Both Moreira et al. (2016) and Estevan et al. (2013) reported similar total response times for the roundhouse kick, defined as the time interval between the stimulus onset and the kick impact. Estevan et al. (2013) reported that RT and kick time were 162 ± 40 ms and 587 ± 90 ms during the roundhouse kick with the feet oriented toward the target. In the current study, the mean premotor times for clenched fist ranged from 149 to 191 ms, while the mean kick times for the round kick following the clenched fist ranged from 560 to 610 ms. These time interval variables align with those reported in previous studies on the round kick (Estevan et al., 2013; Moreira et al., 2016; Sant'Ana et al., 2017). However, a series of combat techniques performed in rapid succession due to tactical requirements allowed us to observe the effects of time requirements on the compilation of action and the initiation of the kicks sequence in both central processing (premotor time) and peripheral processing (kick time). The additional time of 25 to 40 ms in central processing and additional 50 ms required for executing the round kick had a significant impact on the combat outcome. The tactic of stringing together multi-kick movements increases RT and executing time, what is a disadvantage for combat athletes when it comes to attacking or counterattacking.

Taekwondo is one of the combat sports of the Olympic Games, demanding high-intensity physical exertion due to increased mechanical and a physiological loads resulting from frequent amendments to competition rules (Janowski et al., 2021). Previous studies employing the fractionated reaction time approach have investigated the components of central processing and peripheral processing (Ando et al., 2010; Hsu et al., 2023; Kendall et al., 2022). The results indicated that high-intensity exercise shortens premotor time but not motor time. Furthermore, regarding the effects of target height and distance, Estevan and Falco (2013) demonstrated that expert taekwondo athletes had longer RTs when kicking from longer distances, while RTs were similar for the kicks to the chest and the head. Whether the time latency increases during central processing or movement executing, it can lead to disadvantages for the combat athletes. Inevitably, this provides the opponent with more opportunities to attack or counterattack. Consequently, it becomes crucial to develop further game strategies or tactics to reduce potential point losses and enhance kick action efficiency. Numerous studies have examined the influence of kinematics or electromyography by manipulating kick height (Chang et al., 2021; Estevan and Falco, 2013), kick distance (Chang et al., 2021; Falco et al., 2009; Kim et al., 2010), or the kick target (Kim et al., 2017; Wasik et al., 2021) for the round kick. In the present study, utilizing a within-subjects design, we are pioneering in continuously manipulating multi-kick movements to assess their impact on the simple RT and MT of the round kick. Our results serve as evidence base for training and tactics in combat sports. The increase in response complexity within the simple RT paradigm explored in this research offers practitioners insight into understanding the challenges of combat sports and provides an alternative approach for developing a wide variety of experimental designs.

The movements involved in the round kick in the lead foot, the double kick, and the turning kick in this study belong to the round family of kicks. MT measurements shown in Table 1 include the interval of time, known as the return phase, between the end of contact with the target of the prior kick and the foot’s contact with the ground. This interval is longer than the execution period from the toe-off of the ground to contact with the target of the kick alone. The application of these execution times should be made cautiously due to the limitations of the assessment by definition. Further work is required to separate the time interval of the return phase from execution.

## Conclusions

The findings of the present study confirmed the effects of a series of individual kick movements on RT and MTs. It is evident that stringing together multi-kick movements must lead to delays in response programming and kick execution within the simple RT paradigm, even though highly skilled taekwondo athletes demonstrated a greater capacity to perform the round family of kicks in this study. Further research aimed at exploring the MTs of sub-phases and the specifics of kick movements combined in sequences would contribute to a better understanding of the performance deficits in combat sports such as taekwondo.

## Practical Implications

In combat sports, launching consecutive attacks that consist of two or more individual kicks strung together in time is a critical tactic. However, the results of this study confirm that the tactics of multi-kick movements strung together increase RT and execution time, which become disadvantages for attacking or counterattacking in combat athletes. It must be noted that even highly experienced taekwondo athletes required more time to organize and execute sequential kicks. Athletes, coaches, and instructors need to familiarize themselves with the constraints of a series of taekwondo kicks so that they can effectively perform attack or counterattack strategies to gain scores against opponents when increases in time latency occur. This will enable them to counteract the negative effects of slower actions. Finally, the increase in response complexity within the simple RT paradigm in this research provides practitioners with insights into the issues of combat sports and offers an alternative approach for developing a wide variety of experimental designs.
